# Improved transition period management increases milk production of Holstein crossbred cows on smallholder farms in Ethiopia

**DOI:** 10.1007/s11250-026-05032-7

**Published:** 2026-04-17

**Authors:** Oghaiki Asaah Ndambi, Istvan Fodor, Adolfo Alvarez Aranguiz

**Affiliations:** Wageningen Livestock Research, Wageningen University and Research, Wageningen, 6700 AH Netherlands

**Keywords:** Lactation cycle, Milk yield, Strategic interventions, Transition

## Abstract

Enhancing smallholder dairy productivity is critical for improving livelihoods and food security in Ethiopia. This study evaluated the impact of a Lactation Cycle Approach (LCA) intervention, focusing on improved transition cow management, on peak milk yield and projected total lactation milk production. A comprehensive survey was conducted with 2,084 farmers across four major dairy clusters in Ethiopia (Amhara, Northwest Oromia, Southeast Oromia, and Sidama), generating an initial dataset of 198,433 daily milk yield records. Following a rigorous, multi-step quality control protocol, the final dataset for analysis consisted of 100,620 records from 727 Holstein crossbred cows. A linear mixed-effects model revealed that the LCA intervention significantly increased mean peak milk yield in all regions (*p* < 0.001), with gains ranging from + 3.4 kg/day in Amhara to + 4.5 kg/day in Northwest Oromia. No significant differences were found by parity or gender of the farm household head. Using the Wood’s Lactation Curve Model, these increases in peak yield were projected to translate to an estimated additional 700–900 kg of milk per cow over a standard 305-day lactation. The results demonstrate that low-cost, management-focused interventions targeting the early lactation period can significantly increase productivity. The LCA presents a viable strategy for dairy extension services to catalyse professionalisation and economic growth within Ethiopia’s smallholder dairy sector.

## Introduction

Milk production plays a critical role in the livelihoods and food security of smallholder farmers in Ethiopia. However, milk yield remains significantly lower than global averages despite the importance of dairy farming. A key challenge contributing to this low productivity is the lack of knowledge and proper management practices. Many farmers treat all animals uniformly, failing to account for differences in physiological needs across various stages of lactation (Duguna et al. 2016). This generalised approach overlooks the vital transition period, typically three weeks before and three weeks after calving, which plays an important role in determining milk production and overall cow health (Leblanc 2010; Mezzetti et al. 2021).

During the transition period, dairy cows undergo significant changes in their metabolic, hormonal, immune, and nutritional status, and also face major challenges including: (1) a sharp increase in nutrient and energy demand (for fetal growth, colostrum, milk) simultaneous with depressed feed intake (especially around calving); (2) mobilisation of body reserves and risk of metabolic disorders (e.g., ketosis, milk fever, hypocalcaemia, subacute ruminal acidosis, etc.); and (3) immune changes that reduce resistance to infections (Dewulf et al. 2024; Magro et al. 2024; Mushfiq et al. 2004; Tufarelli et al. 2024; Redfern et al. 2021).

Proper management during the transition period, including providing adequate feed and water, as well as stress reduction, has been shown to improve milk yield, health, and reproductive performance, while reducing the incidence of metabolic disorders (Grummer 1995; Sordillo and Raphael 2013; Tapp et al. 2025). Despite its well-documented importance, basic transition period management practices are often overlooked in Ethiopia’s smallholder systems, where resources and training are limited.

One of the largest benefits of transition period management is its impact on peak milk production, which typically occurs within the first few weeks after calving. Research has shown that improvements in peak production during early lactation can significantly increase overall milk yield across the full lactation period (Bauman and Currie 1980; Roche et al. 2013). In smallholder systems, achieving even modest gains in peak production can translate into substantial increases in total milk production, improving household income and food security. These improvements have the potential to deliver far-reaching benefits, which will not only improve the livelihoods of individual households but also strengthen the broader dairy industry in Ethiopia, a cornerstone of rural development and food security.

By focusing on cost-effective, evidence-based interventions during the transition period, this study aims to demonstrate how smallholder farmers in Ethiopia can increase dairy production by adopting basic management practices. This study was conducted under farmer-managed conditions where farmers had the choice to decide on interventions to adopt from a list of improvement options, and this varied from farmer to farmer, as opposed to controlled experimental settings. Additionally, by integrating a quality control methodology, the study seeks to establish a systematic approach for monitoring, ensuring consistency and improving data collected from a smallholder setting where data quality is often an issue.

## Methods

### Study area and population

**Fig. 1:**
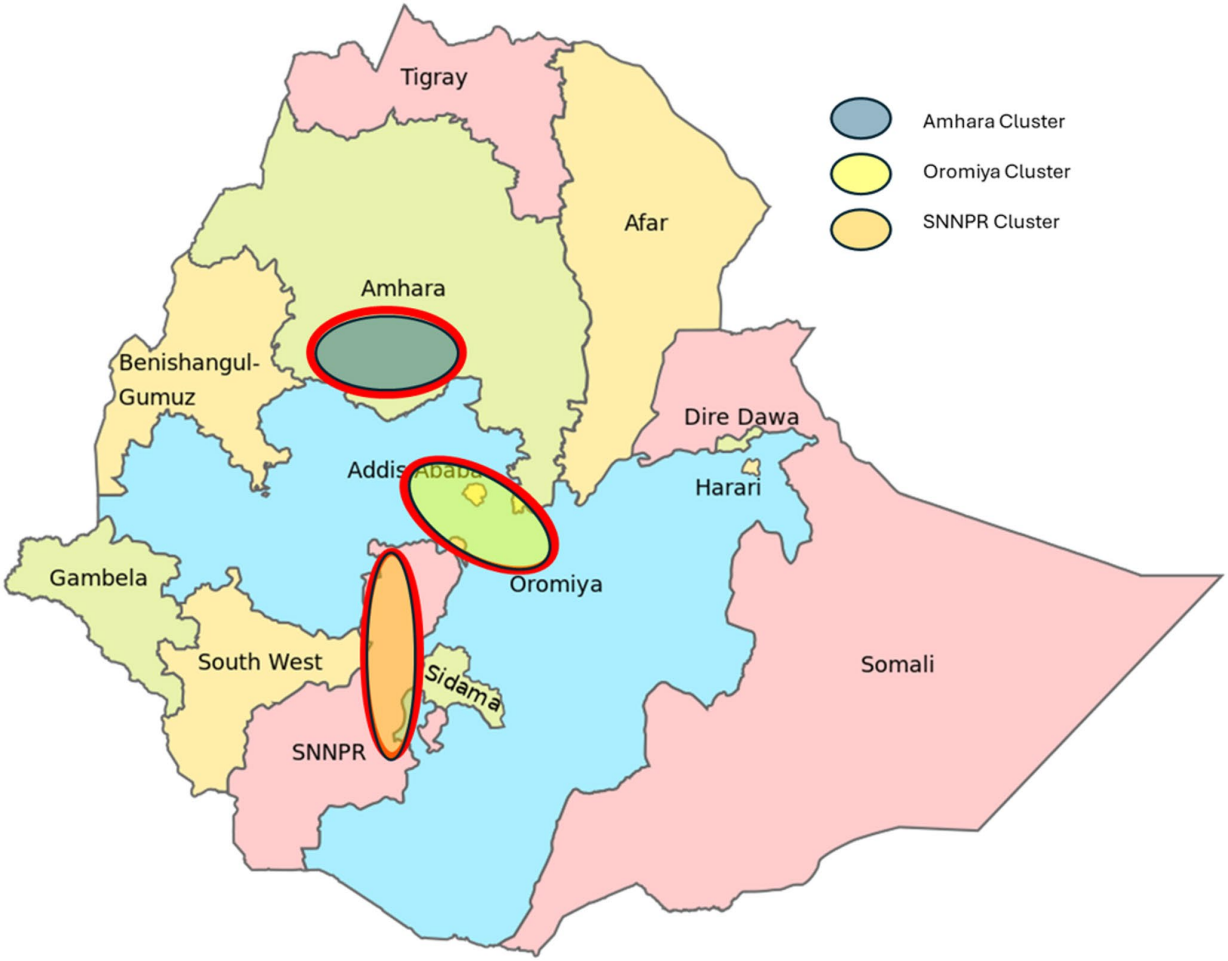
Dairy clusters showing district borders

The study was conducted across three key dairy clusters in Ethiopia, including Oromia, Amhara, and the Southern Nations Nationalities and Peoples Region (SNNPR; Fig. 1). These regions were selected due to their significant involvement in dairy farming and the substantial number of smallholder farmers managing crossbreed cows, as noted in a study by Ndambi et al. (2021).

### Selection of farmers

The farming households for this study were selected based on the following criteria: (a) having at least one crossbreed heifer/cow in rural households and at least 2 in urban households, (b) having and willing to avail land for fodder production, and (c) living at a permanent residence. Additional factors such as motivation, gender balance, and the proportion of milk sold were also considered during selection.

The first cohort of 2,847 farmers, managing 3,506 pregnant cows, was organised into 300 Dairy Farm Innovation Groups (DFIGs) to facilitate the delivery of advisory services related to selected practices. This initiative was further enhanced through the integration of digital platforms. A robust network of 135 service providers, including agro-input dealers (AgIDs), cooperatives, development agents (DAs), artificial insemination (AI) technicians, veterinarians, and other experts, was carefully selected, trained, and deployed to ensure effective implementation of the practices (Bezabih et al. 2023a, b).

### Study design and limitations

This study used a before–and–after, herd-level evaluation to assess the effect of introducing an improved transition management program under commercial farm conditions. Farms were offered a package of recommended transition‑management practices; however, not all farms implemented all practices, and adoption intensity varied among farms. Because implementation occurred in real farm settings, the study compares herd‑level performance before and after the program’s introduction, rather than evaluating individual management components. A conventional control group was not included, as withholding recommended management improvements was not ethically or practically feasible.

Implementation fidelity, defined as the extent to which specific practices were adopted and adhered to, was not measured systematically. As a result, the intervention is evaluated as an integrated management package with variable uptake, rather than as a uniform protocol applied equally across farms. Additionally, farms and cows with incomplete or inconsistent records were excluded during data quality control, which may introduce selection bias toward farms with more reliable record‑keeping.

These design features should be considered when interpreting results. A more detailed discussion of adoption variability, potential confounding factors, and implications for causal inference is provided in the discussion.

### Practices introduced

The selection of dairy practices for implementation was based on a set of carefully defined criteria. Firstly, the practices were chosen for their ability to rapidly improve milk production once implemented. Secondly, these practices needed to be straightforward for the farmers to apply, ensuring they could be easily integrated into existing dairy operations. Thirdly, the practices needed to be easily explainable by the advisors and comprehensible to farmers, facilitating smooth adoption and implementation. Finally, the selected practices were required to involve little to no additional cost, making them economically viable for widespread use (Table 1).

**Table 1:** Description of selected practices

Practice	Description
Water supply	Increase the number of times and volumes of water supplied to cows
Forage supply	Increase forage supply volumes
Smart feeding	Prioritize high-quality feeds according to the lactation stage of cows
Aeration and lighting	Open barn sides to improve aeration and lighting
Space and resting areas	Provide enough lying space and improve bedding conditions

Farmers were provided with comprehensive knowledge of various aspects related to these practices to help them understand their underlying principles. The training focused on several key areas, like the importance of water provision by increasing supply frequency to ensure ad libitum access, advanced feeding strategies, such as providing ad libitum forage, high-quality forage and concentrate distribution according to lactation stages, ensuring that nutritional needs are met throughout the lactation cycle as well as effective body condition management and monitoring of other key cow signals. Enhancements to barn facilities were discussed to improve cow comfort, including better lighting, ventilation, space, and resting areas.

Farmers were trained and encouraged to adopt all the above-mentioned practices. However, they were free to select and adopt any of those they preferred, mainly based on the availability of financial and labour resources. Each of the farmers in the intervention group adopted at least three of the five practices, and they had full control over their management decisions.

### Data collection

The data collection process included a comprehensive survey conducted with 2084 farmers across key dairy clusters in Ethiopia following the implementation of the Lactation Cycle Approach (LCA), including daily milk production (morning and afternoon milking) recorded by the farmers in a data collection card designated for this purpose, together with previous lactation peak production. With this approach, increasing the peak of the lactation curve was targeted, and it was hypothesised that this increase would lead to an increase in total milk production of the cow for the full lactation.

From the survey, an initial dataset was generated comprising 198,433 daily milk yield records from 1,970 cows managed by 1,776 farmers. The distribution of farmers, number of cows, and mean cows per farmer (in parentheses) by dairy clusters was as follows: Amhara: 369 (412; 1.12), Northwest Oromia: 467 (534; 1.14), Southeast Oromia: 498 (537; 1.06), and Sidama: 442 (487; 1.10). Households were classified based on whether they were male or female-headed.

The data was collected on various aspects of dairy farming, including milk production, management practices, and the adoption of improved techniques. Farmers recorded daily milk yields from both morning and afternoon milkings, allowing for continuous monitoring of production trends. They were supported by service providers who visited the farms once a month and carried out a quality check, in addition to consolidating the data. Specifically, the analysis centred on milk yield per cow during the first 100 days of lactation and peak milk production. Milk production was assessed by evaluating changes in peak yield compared to the previous lactation season, providing a clear measure of the impact of management interventions.

### Data quality control

**Fig. 2:**
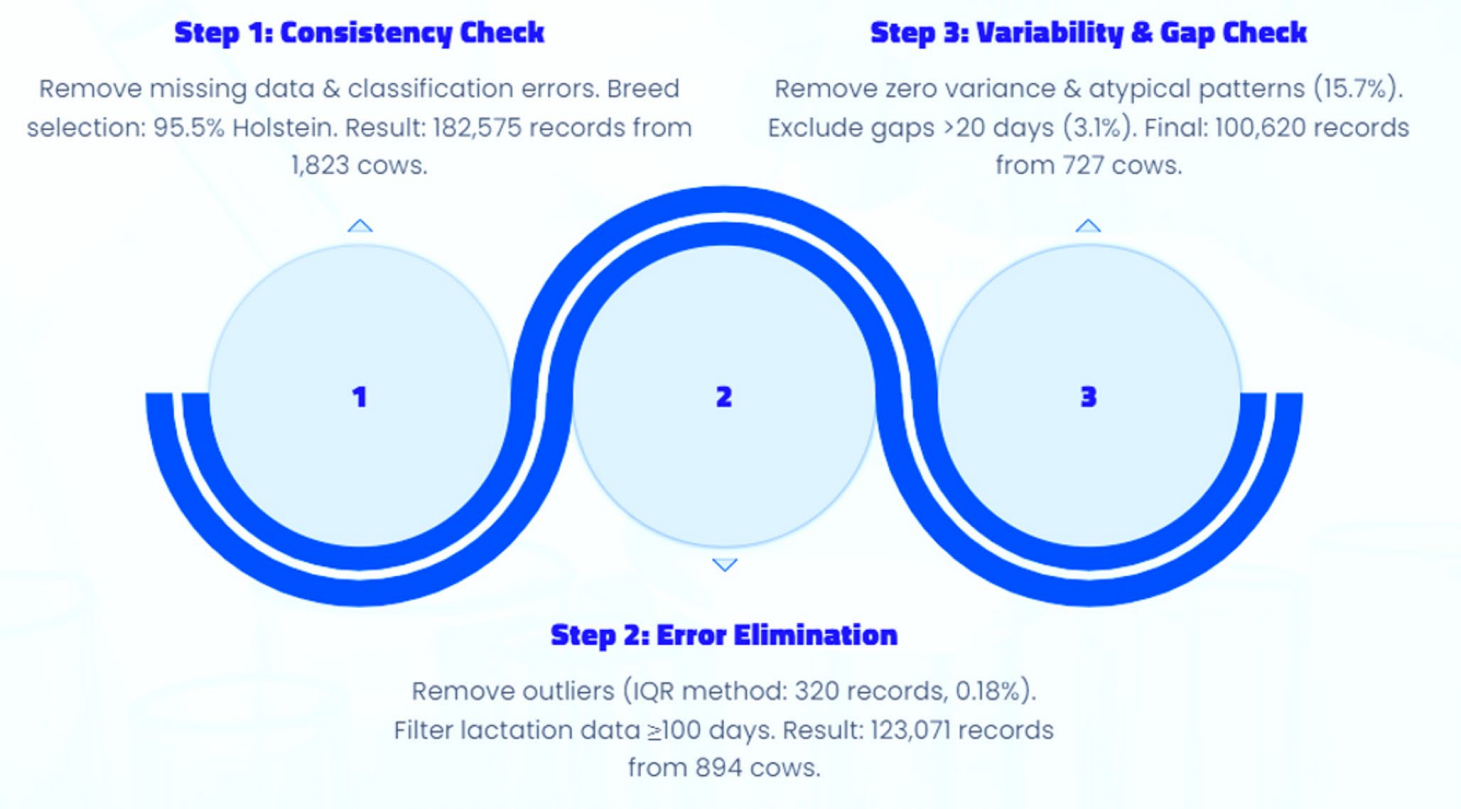
Data quality control process summary

A rigorous quality control process was applied to ensure the inclusion of high-quality data in subsequent analyses (Fig. 2). Farms and cows with incomplete or inconsistent records were excluded during data quality control to ensure reliable analyses. This may have resulted in retaining farms with better record‑keeping, introducing potential selection bias. The implications of this limitation are addressed in the discussion.

#### Step 1: General consistency check

Before quality control, 198,433 daily milk yield records from 1,970 cows were available in the dataset. Two cows with missing household information were removed. Additionally, seven cows lacking crossbreed classification (Holstein or Jersey crossbred) were excluded. Since 95.5% of the remaining cows (*n* = 1,873) were Holstein crossbreds and only 4.5% (*n* = 88) were Jersey crossbreds, further analyses focused solely on Holstein crossbreds. Next, 48 cows were excluded due to non-zero milk yield recorded in their previous parity as heifers, and two cows were removed due to missing parity information. Cows were categorised by parity (1, 2, 3+, representing the first, second, and third or higher parity, respectively), with 145 cows in parity 1,383 cows in parity 2, and 1,295 cows in parity 3 or higher. After the general consistency check, a total of 182,575 morning, evening, and daily total milk yield records from 1,823 cows were retained.

#### Step 2: Further elimination of data recording errors or outliers

To ensure the accuracy of daily total milk yield, outliers in morning, evening, and total yield records were removed using the criterion of values below the first quartile minus three times the interquartile range (IQR) or above the third quartile plus three times the IQR. This step eliminated 320 records (0.18% of the total). As peak milk yield is typically observed around 60 days in milk (DIM) (Madalena et al. 1990; Galukande et al. 2013), only cows with data for at least 100 days of lactation were retained, resulting in the removal of 32.5% of records and leaving 123,071 records from 894 cows.

#### Step 3: Final variability check

To further minimise potential reporting errors, cows were removed if the variance of any of their morning, evening, or daily total milk yield time series was zero (i.e., all values identical) or fell outside the 5th − 95th percentile range of non-zero variances (i.e., atypical variance). This step excluded an additional 15.7% of the remaining records, leaving 103,794 records from 751 cows. Furthermore, cows were removed if they had a gap in milk yield records exceeding 20 consecutive days (3.1% of cows). The final dataset consisted of 100,620 records from 727 cows. Peak milk production (kg/day) for the current lactation was defined as the highest recorded daily total milk yield for each cow.

### Data analysis

All statistical analyses were performed in R version 4.3.1 (R Core Team, 2023). The dataset was subjected to a linear mixed-effects model, using the lme4 package (Bates et al. 2015). First, a full model was fitted, with peak milk yield (kg/d) as the dependent variable, parity (1, 2, and 3+), intervention (two levels: before/after), household (two levels: male- or female-led), and all their two-way interactions as fixed effects, and cow as a random effect. Non-significant interactions were sequentially removed, and the model was refitted. Pairwise comparisons of the parity levels were performed with Tukey’s post hoc comparisons. The level of significance was set to 0.05.

### Projected lactation curves and estimated total milk yield

To better understand the implications of observed changes in peak milk yield across regions, the Wood’s Lactation Curve Model (Wood 1967) was applied to project complete 305-day lactation curves for each region before and after the implementation of the LCA. This model provides an empirical representation of milk production dynamics throughout the lactation period, defined as *Y (t) = at*^*b*^*e*^*−ct*^, where *Y (t)* represents milk yield (kg/day) at time 𝑡, and parameters 𝑎, 𝑏, and 𝑐 describe the initiation, rise, and decline phases of lactation, respectively.

Using the mean peak milk yields recorded in each region as anchor points, the model estimated full lactation curves that illustrate both the temporal distribution of milk production and total projected yield.

## Results

The number of farmers and cows (in parentheses) analysed for management improvement program impact by region and gender of household head is shown in Table 2.

The data quality control led to a retention of data from 713 farms and 727 cows, from an original 1776 farms and 1970 cows, showing a retention rate of 40% of farms and 37% of cows. These rates varied across the clusters, with South East Oromia having half of its cows retained, while only one-fifth of the cows in Sidama were retained.

From the total sample of farmers, about 85% were males and only 15% were females, with Amhara having the lowest proportion of female-headed farms (9%) and Sidama with the highest proportion (22%). As the study focused on transition period interventions, there were no heifers in the “after intervention” group. Most cows in the study were in their 3rd lactation and above, which almost doubled from 39% of the total sample before to 74% after, as all cows in their 2nd lactation before were now in their third lactation.

**Table 2:** Number of farmers (and cows) retained for data analysis after data screening

Dairy cluster		Amhara		NWO		SEO		Sidama		Total
	Parity	Female HHH	Male HHH	Female HHH	Male HHH	Female HHH	Male HHH	Female HHH	Male HHH	
Before intervention	Heifer	-	2 (2)	-	2 (2)	3 (3)	20 (20)	3 (3)	17 (18)	47 (48)
	1	3 (3)	27 (28)	4 (4)	33 (33)	9 (9)	43 (43)	5 (5)	14 (15)	138 (138)
	2	4 (4)	44 (44)	9 (9)	49 (49)	18 (18)	78 (78)	11 (11)	35 (36)	248 (249)
	3+	6 (6)	55 (56)	9 (9)	101 (106)	16 (17)	77 (80)	3 (3)	13 (13)	280 (286)
Total		13 (13)	128 (130)	22 (22)	185 (190)	46 (46)	218 (217)	22 (22)	79 (78)	713 (722)
After intervention	1	-	2 (2)	-	2 (2)	3 (3)	20 (20)	3 (3)	17 (18)	47 (48)
	2	3 (3)	27 (28)	4 (4)	33 (33)	9 (9)	43 (43)	5 (5)	14 (15)	138 (140)
	3+	9 (10)	99 (100)	18 (18)	148 (155)	34 (35)	154 (158)	14 (14)	47 (49)	523 (539)
Total		12 (13)	128 (130)	22 (22)	183 (190)	46 (47)	217 (221)	22 (22)	78 (82)	708 (727)

NWO = North West Oromia; SEO = South East Oromia; HHH = household head

**Fig. 3:**
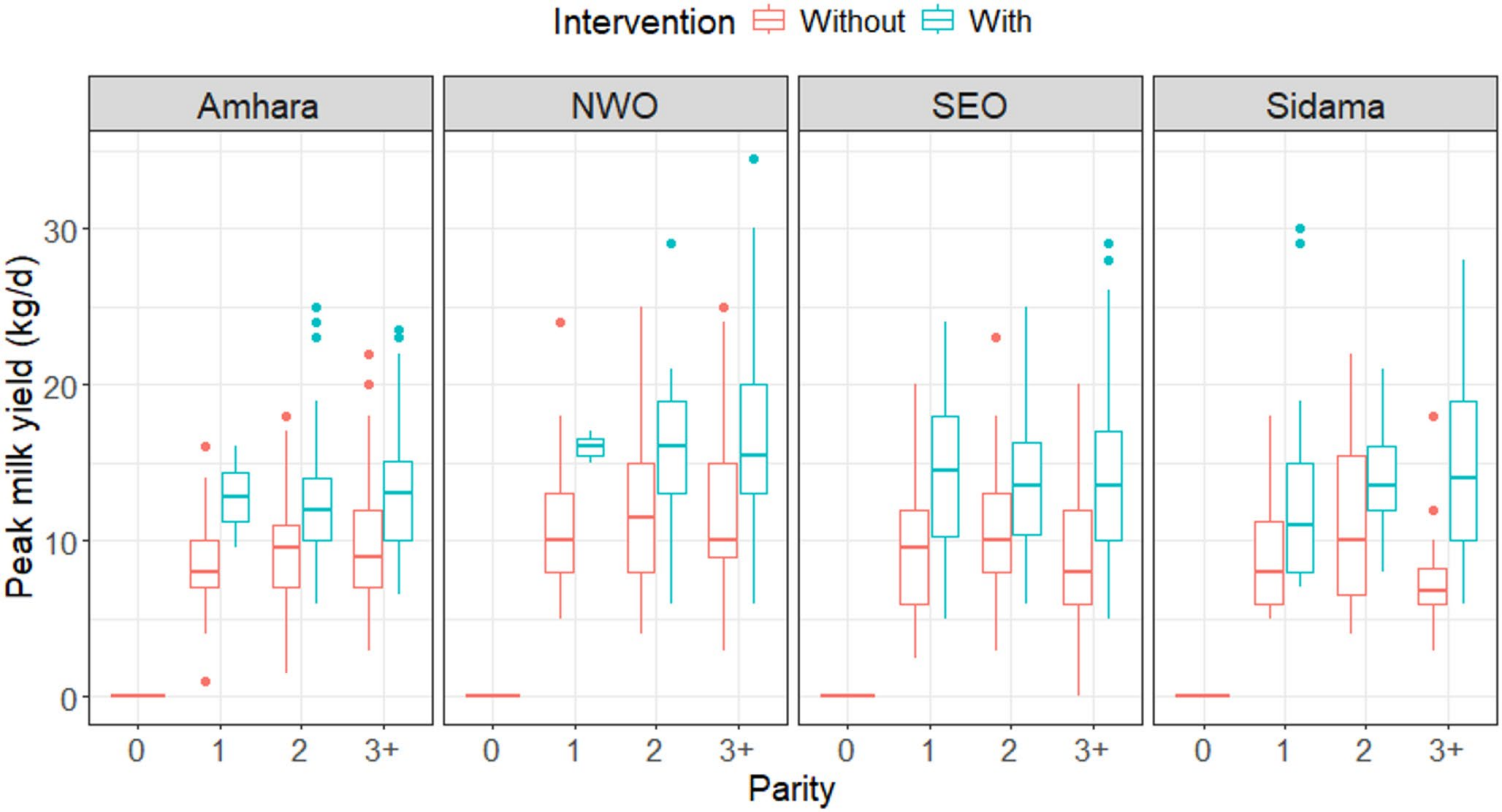
Descriptive plot of peak milk yield (kg)

The peak milk yield for the cows before (without) and after (with) the intervention is shown by region and parity in Fig. 3. In all four regions, and for all parity numbers, the median peak milk yield was higher in the cows after the intervention compared with the peak before the intervention. In most cases, the interquartile range of the peak milk yield was wider in the cows after the intervention compared to the situation before the intervention. Before the intervention, the median peak milk yield was lowest in parity 3 + for all regions except Amhara. After the intervention, the median peak milk yield was more similar across the different parities in all the regions except Sidama, where the median peak milk yield was highest in the cows with parity 3 + and lowest in the cows with parity 1.

Table 3 shows the peak milk yields by parity and by gender of the household head. There were no differences in mean peak milk yield by parity (*P* = 0.153) and by gender of household head (*P* = 0.607, Table 3).

**Table 3:** Peak milk yield by parity and household head

		Mean peak milk yield (kg/d)	SE	P-value
Parity	1	12.0	0.34	0.153
	2	12.3	0.27	
	3+	12.6	0.27	
Household	Female-headed	12.4	0.45	0.607
	Male-headed	12.2	0.20	

The highest peak milk yields were observed in North West Oromia before as well as after the interventions (Table 4). We found a significant interaction between intervention and region (*P* = 0.003), meaning that the impact of improved transition cow management on peak milk yield depended on the region. Peak milk yield increased in each region after the management changes, with the largest increase in North West Oromia (+ 4.5 kg, SE: 0.20), followed by South East Oromia (+ 4.3 kg, SE: 0.19), Sidama (+ 4.2 kg, SE: 0.32), and Amhara (+ 3.4 kg, SE: 0.24).

**Table 4:** Peak milk yield by region and intervention

Region	Intervention	Mean peak milk yield (kg/d)	SE	*P*-value
Amhara	Before	9.6	0.44	< 0.001
	After	13.0	0.45	
NWO	Before	11.6	0.38	< 0.001
	After	16.1	0.39	
SEO	Before	9.7	0.33	< 0.001
	After	14.0	0.34	
Sidama	Before	10.2	0.49	< 0.001
	After	14.3	0.48	

**Fig. 4:**
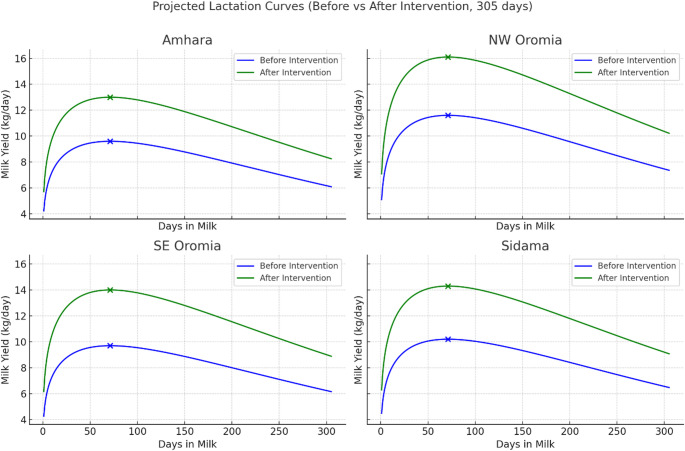
Projected lactation curves (305 days) before and after LCA implementation across dairy clusters in Ethiopia

Results for the projected lactation curves indicate that the LCA intervention substantially increased projected total lactation yield across all clusters, consistent with the observed rise in peak milk yield (Fig. 4). Specifically, increases in mean peak yield ranged from 3.4 to 4.4 kg/day between the pre- and post-intervention periods, corresponding to an estimated increase of approximately 700–900 kg of milk per cow per lactation (assuming 305 days).

## Discussion

This study provides robust evidence that the Lactation Cycle Approach (LCA) significantly improved peak milk yield across all major Ethiopian dairy clusters. By aligning management interventions with the physiological stages of lactation, the LCA effectively increased productivity among smallholder farmers operating under diverse agroecological and management conditions. These results conform to the results of other studies in Ethiopia, where various changes in management significantly changed the milk yield of Holstein Friesian crossbred cows (Bedada et al. 2021; Birhan et al. 2023; Hatew et al. 2023).

The findings confirm that strategic interventions during the transition and early lactation periods can markedly increase peak milk yield, with absolute gains ranging from 3.4 to 4.5 kg/day. Other studies in Ethiopia have also indicated an increase in peak milk yield through strategic interventions (Amejo et al. 2024; Bezabih et al. 2023a, b). A study by Bateki et al. (2021) in Kenya shows that these gains could be enhanced by using information and communication technology applications. Using Wood’s lactation curve model (Wood 1967) to project total yields, these improvements correspond to approximately 700–900 kg of additional milk per cow per lactation, underscoring the potential of targeted management practices to raise household income and strengthen farm resilience. An increase of 700 kg of milk translates to a milk income of ETB 28,000 (approx. USD 390) per lactation based on the minimum price of ETB 40 (USD 0.55) offered by producers’ cooperatives in 2024 (Fita and Fekata 2024).

These modelled projections provide additional evidence that early lactation interventions not only enhance short-term production but also yield sustained improvements over the full lactation period, confirming the effectiveness of the LCA in improving both biological performance and management efficiency among smallholder dairy farmers.

The significant region-by-intervention interaction (*p* = 0.003) indicates that the LCA’s impact varied by production environment. The largest gains in Northwest Oromia likely reflect improved genetic, stronger extension linkages, better input access, and greater farmer engagement. These regional differences highlight the importance of tailoring extension approaches to local contexts rather than applying a uniform model.

Unexpectedly, cow parity did not significantly affect peak yield (Table 3). This is, however, contradictory to findings by Begna et al. (2023) and Senbete and Abebe (2021), who noted a significant difference in milk yield for different cow parities in Ethiopia. This study’s findings suggest that the LCA mitigated age-related performance gaps by improving nutritional and management support during early lactation. Similarly, no significant gender-based differences were observed, demonstrating that the approach is inclusive and effective across household types.

The wider yield variability observed after intervention reflects differences in adoption intensity, a typical feature of farmer-led programs. This variation underscores the need for greater consistency in applying LCA protocols to achieve more uniform productivity gains.

We evaluated the transition‑management program as an integrated package under commercial conditions and did not quantify implementation fidelity (i.e., which practices each farm adopted or the degree of adherence). Adoption intensity likely varied across farms; thus, we report herd‑level effects only. While specific compliance metrics for each LCA practice were not recorded, our analytical approach reflects an Intention-to-Treat (ITT) framework. This ensures that the results represent the real-world effectiveness of the intervention within a complex farming system, rather than just its efficacy under perfect adherence. We acknowledge that the observed increase in post-intervention variability may indeed stem from varying degrees of partial adoption. The consistent direction and timing of responses after implementation support effectiveness, but we did not isolate the contribution of specific components. Future studies should incorporate structured fidelity assessments (e.g., checklists, direct observations, or digital logs) to quantify adoption and identify the most impactful practices.

Our before–and-after design shows strong, biologically plausible associations between program implementation and increased milk production. However, the absence of a formal control group limits definitive causal inference, and unmeasured confounding (e.g., seasonal effects, concurrent farm changes, or additional advisory inputs) cannot be fully excluded. We therefore interpret the results as associations consistent with a causal effect, with appropriate caution.

During data quality control, some farms and cows were excluded for incomplete or inconsistent records, which may introduce selection bias. Retained farms likely had better record‑keeping and possibly higher baseline management capacity, potentially affecting the magnitude (but not the direction) of estimated effects. More complete data likely improved precision; farms with poorer records may have exhibited similar trends with greater variability. Accordingly, effect sizes may be modestly conservative or slightly inflated, but the overall direction of the findings appears robust. Despite rigorous data cleaning, the high attrition rate (retaining 37% of the initial data) illustrates persistent challenges in farmer-recorded datasets, including estimation errors and limited literacy. Future research should strengthen data systems through digital tools and continuous farmer training.

Overall, the LCA represents an innovative, scalable, and low-cost extension methodology that leverages farmers’ responsiveness to short-term, visible results while increasing long-term productivity. The approach could be used to predict milk yield deviation from the expected values and support transition management (Hannon et al. 2024). Integrating the LCA into national extension frameworks could substantially improve dairy performance in Ethiopia and beyond, promoting sustainable intensification and improved livelihoods for smallholder farmers.

In conclusion, milk yield and income generation from smallholder dairy farms in Ethiopia can be increased significantly by targeting low-cost interventions leading to an upward shift in the lactation curve. Extension systems for low-income farmers could use the lactation curve approach as a starting point for dairy development through professionalisation of dairy production, which could encourage farmers to invest more once they start seeing the benefits from smaller investments.

## Data Availability

Data will be made available on reasonable request.
